# Chromosomal resistance mutations facilitate acquisition of multidrug-resistant plasmids in Escherichia coli

**DOI:** 10.1099/mic.0.001599

**Published:** 2025-09-25

**Authors:** Khadija-Siddiqa N. Hanga, Michael A. Brockhurst, Michael J. Bottery

**Affiliations:** 1 Division of Evolution, Infection and Genomics, School of Biological Sciences, Faculty of Biology, Medicine and Health, University of Manchester, Manchester, M13 9PT, UK

**Keywords:** carbapenemase, extended-spectrum *β*-lactamase (ESBL), fitness, multidrug resistance, plasmid, synergistic resistance

## Abstract

Bacteria can gain multiple resistance mechanisms in a single step by the acquisition of multidrug-resistant (MDR) plasmids, but it is unclear how antibiotic selection during the acquisition of MDR plasmids affects the evolution of additional resistance mechanisms. Through conjugating separate extended-spectrum *β*-lactamase (ESBL)- and carbapenemase-producing MDR plasmids into plasmid-naive *Escherichia coli* hosts, we examine the effects of acquisition of a single plasmid or co-acquisition of multiple plasmids upon fitness costs, resistance and subsequent genomic adaptation. We show that acquisition of pOXA-48, encoding OXA-48 carbapenemase, is associated with highly variable fitness costs and levels of resistance to ertapenem in transconjugants independent of the presence of pLL35. This phenomenon was not observed during the acquisition of ESBL CTX-M-15–encoding pLL35 alone. Within a single growth cycle, transconjugants receiving pOXA-48 rapidly gained parallel mutations affecting the membrane porin OmpF, or its regulators OmpR or EnvZ. These chromosomal mutations were not compensatory for the fitness costs imposed by the plasmid, nor did they provide significant increases in resistance to carbapenems in the absence of the pOXA-48. Rather, they acted synergistically with the plasmid-encoded carbapenemase, which alone only provided marginal resistance, together providing high-level resistance to ertapenem. Such rapid evolutionary processes may play an important role in plasmid dynamics within environments with strong antibiotic selection for plasmid-encoded antimicrobial resistance genes (ARGs), particularly when these ARGs provide only marginal resistance.

## Data Availability

The sequencing data generated in this study have been deposited in the European Nucleotide Archive database under accession no. PRJEB90055. Individual sample accession numbers are included in Table S6.

## Introduction

Antibiotic resistance is a growing public health crisis, with an estimated 1.27 million deaths attributed to antibiotic-resistant infections in 2019 alone [1]. Resistance mechanisms in bacteria can be intrinsic, due to inherent structural and functional traits, or acquired through mutations or horizontal gene transfer (HGT) of antimicrobial resistance genes (ARGs) encoded on mobile genetic elements (MGEs) [2,3]. MGEs can promote the accumulation and rapid spread of multiple resistance mechanisms in a single step. When bacteria acquire MGEs encoding ARGs, antibiotics may become ineffective, allowing strains to grow in the presence of otherwise lethal antibiotic concentrations and pose a significant challenge to treatment [3].

In recent decades, the rise of carbapenem-resistant Enterobacteriaceae (CRE) and extended-spectrum *β*-lactamase (ESBL)-producing Enterobacteriaceae has been particularly concerning. Infections caused by these antibiotic-resistant bacteria, especially in intensive care units and among patients with severe underlying conditions, are associated with high rates of morbidity and mortality [2,4, 5]. Resistance within CRE is primarily spread through HGT mediated by conjugative plasmids encoding carbapenem-hydrolysing carbapenemase enzymes [6,9]. Other mechanisms, such as porin mutations and efflux pump overexpression, can also confer resistance to carbapenems, particularly when accompanying *β*-lactamase enzymes [5,10].

Although Enterobacteriaceae, such as *Escherichia coli* and *Klebsiella pneumoniae*, form part of the healthy human gut flora, they are also frequently associated with human and animal infections. Invasive illnesses caused by multidrug-resistant (MDR) lineages of Enterobacteriaceae pose a significant barrier to successful treatment. Such invasive disease-causing lineages are often associated with the acquisition of one or more MDR plasmids [11]. Plasmids carried by MDR strains frequently encode ESBLs, and due to the increase in the usage of carbapenems to treat infections, the co-occurrence of separate carbapenem resistance plasmids is on the rise [3,11]. Combatting such increasing rates of MDR Enterobacteriaceae infections requires understanding of how the carriage of multiple MDR plasmids affects bacterial fitness and resistance levels.

Whilst acquiring an MDR plasmid can provide significant fitness advantages during antibiotic treatment, plasmids often impose a fitness cost in the absence of antibiotics. Such fitness costs can arise through multiple different and often interacting mechanisms, including dysregulation of metabolic pathways, producing and accumulating toxic products and altering gene expression [12,14]. The carriage of multiple plasmids can have complex effects on fitness costs. Positive epistasis between plasmid costs, whereby the cost of acquiring additional plasmids is less than the sum of the additive costs imposed by each individual plasmid, can promote the accumulation of multiple plasmids within single cells [13,15]; however, positive epistasis is not universally observed [16]. The balance between the cost imposed by an MDR plasmid in the absence of antibiotic selection and the benefit in its presence is a critical factor in the persistence of resistance plasmids within bacterial populations [13,17]. However, fitness costs associated with plasmids can be ameliorated by compensatory mutations either on the chromosome, plasmid or both, stabilizing plasmids within bacterial lineages [4,18,20], and can occur extremely rapidly, even during the outgrowth of transconjugant colonies [21]. Moreover, several experimental studies report that single compensatory mutations can ameliorate the costs of multiple distinct plasmids [16,20], favouring the maintenance of multiple plasmids within lineages.

Here, we examine the immediate effects of coinfection by two clinical conjugative plasmids, pLL35 and pOXA-48, on resistance, fitness costs and compensatory mutations that arise within a single growth cycle after receiving the plasmids. The 106 kb MDR IncFII(K)-9 group plasmid pLL35 was originally isolated from *K. pneumoniae* and encodes the ESBL CTX-M-15 conferring resistance to cefotaxime (CEF) which is the most commonly occurring ESBL worldwide [22,23]. pLL35 is highly stable due to its toxin/antitoxin stability systems [16,24]. The 65 kb IncL group pOXA-48 plasmid was isolated from a separate *K. pneumoniae* infection and encodes the carbapenemase OXA-48, which accounts for 52% of carbapenem resistance cases referred to the UK AMRHAI Reference Unit in 2018 [14,25]. We measured resistance to CEF or ertapenem (ERT) and growth kinetics without antibiotics for *E. coli* MG1655, either singly or coinfected with pLL35 and pOXA-48, and obtained whole-genome sequences for multiple independent transconjugants, a single 24 h growth cycle after receiving the plasmid. We found that the acquisition of pOXA-48, either alone or together with pLL35, was facilitated by chromosomal resistance mutations affecting genes encoding outer membrane proteins. Using Keio single-gene knockout strains, we show that the mutated chromosomal loci are not classical compensatory mutations but instead act synergistically with the plasmid’s OXA-48 ARG to provide high-level resistance to ERT.

## Methods

### Bacterial strains, plasmids and culture conditions

Two isogenic strains of *E. coli* K12 MG1655 chromosomally labelled with EGFP (MG1655::GFP; subsequently denoted MG1655G) or mCherry (MG1655::mCherry subsequently denoted MG1655R) at the attB natural insertion site through λ red homologous recombination were used as recipients for conjugation experiments unless otherwise stated [19]. Fluorescently labelled *E. coli* MG1655 strains were also labelled with kanamycin resistance (NeoR). The plasmid donors used for conjugations were *E. coli* J53 for pOXA-48 and *E. coli* MG1655 for pLL35, both established by conjugation from their natural *K. pneumoniae* hosts (Table S1, available in the online Supplementary Material).

All strains were cultured in liquid Nutrient Broth (NB) (Oxoid) at 37 °C, shaken at 180 r.p.m., in 6 ml media in 50 ml Falcon tubes or on Nutrient Agar (Oxoid) for culture on solid media. Resistance profiles of all strains were confirmed by plating onto solid media containing kanamycin (25 µg ml^−1^) for fluorescent recipients, sodium azide (100 µg ml^−1^) for *E. coli* J53, CEF (4 µg ml^−1^) for strains containing pLL35 or ERT (0.5 µg ml^−1^) for strains containing pOXA-48.

### Plasmid conjugation

Twenty-four independent conjugation assays were conducted to construct eight biological replicates of MG1655 pLL35, MG1655 pOXA-48 and MG1655 pLL35+pOXA-48 transconjugants. The pLL35 and pOXA-48 resistance plasmids were transferred individually into *E. coli* MG1655 via conjugation. Overnight cultures of recipients (MG1655G and MG1655R) and donors (MG1655 pLL35 and J53 pOXA-48) were inoculated in 5 ml of NB from single colonies isolated from selective plates. pLL35 was conjugated into recipients through liquid conjugation, and donor and recipient overnight cultures were mixed at ratios of 1 : 3 for pLL35 and incubated at 37 °C in non-selective NB without shaking for 24 h. pOXA-48 conjugation was conducted on solid agar, donor and recipients were mixed at a 1 : 3 ratio and 50 µl of the mixture was added on sterile filter papers placed on non-selective nutrient agar plates and incubated at 37 °C for 24 h. To create the double plasmid-bearing *E. coli* MG1655 pLL35+pOXA-48, a second round of conjugation was performed using fluorescent *E. coli* MG1655::GFP pOXA-48 and *E. coli* MG1655::mCherry pLL35 at a 1 : 1 ratio. Transconjugants were selected by plating serial dilutions of the mixed cultures onto nutrient agar supplemented with either CEF, ERT or both. All transconjugants were validated using a multiplex PCR assay designed to amplify the backbone of the two MDR plasmids and their resistance genes separately (Table S2). Eight independent transconjugant colonies from each condition were picked into antibiotic-free NB and grown overnight at 37 °C with shaking, totalling 24 transconjugants. Six hundred microlitres of each culture were then mixed with 400 µl of sterile glycerol and stored at −80 °C. All subsequent experiments were conducted using these eight independent validated transconjugants.

### Plasmid stability assay

To evaluate the stability of pLL35 and pOXA-48 plasmids, all the transconjugants were passaged for 10 days in the absence of antibiotic selection, and the maintenance of the plasmids was tracked by selective plating. Glycerol stocks of each of the 24 transconjugants were revived by streaking onto the appropriate selective plates. Single colonies were picked into 5 ml antibiotic-free NB liquid media and incubated overnight at 37 °C, 180 r.p.m. (T₀). Ten consecutive daily passages were conducted by transferring 5 µl of culture into 5 ml of fresh antibiotic-free NB. For each daily transfer, the density of plasmid-containing and plasmid-free cells within the cultures was calculated by plating serial dilutions onto non-selective and selective agar plates (pLL35: CEF 4 µg ml^−1^; pOXA-48: ERT 0.5 µg ml^−1^; pLL35+pOXA: CEF 4 µg ml^−1^+ERT 0.5 µg ml^−1^).

### Growth curves

Growth curves were conducted to evaluate the growth kinetics of the newly constructed plasmid-bearing strains and the isogenic parental plasmid-free strain. Each of the eight transconjugants from each plasmid condition was individually streaked from glycerol stocks onto the appropriate antibiotic selection plates and incubated overnight at 37 °C. The plasmid-free parental strains were also cultured on nutrient agar plates. Three individual replicate colonies were picked from each growth plate with sterile cocktail sticks into 96-well plates containing 200 µl of NB, resulting in three technical replicates of each of the eight biological replicates, which were incubated overnight at 37 °C. The saturated overnight cultures were diluted to an OD (OD_600_) of 0.05 in fresh NB in a 96-well plate. This was incubated at 37 °C with shaking at 180 r.p.m., with a 3 mm orbital radius for 24 h in a Tecan Infinite M200 plate reader. The OD_600_ was measured every 20 min, and growth parameters of lag time, maximum growth rate, maximum OD_600_ and area under the curve (AUC) were determined using the ‘ggplot2’ and ‘dplyr’ packages in RStudio 2022.07.2.

### MIC assay

MIC assays for CEF and ERT were performed using the broth microdilution method described by Wiegand *et al*. [26]. Transconjugants were streaked onto the appropriate selective agar plates and grown overnight at 37 °C. Three separate colonies of the parental plasmid-free strain and each of the eight pLL35, pOXA-48 and pLL35+pOXA-48-harbouring transconjugants were then picked into 5 ml of NB from the selective agar plates and grown overnight at 37 °C with shaking at 180 r.p.m., resulting in three technical replicates of each of the eight biological replicates. Overnight cultures were diluted to 5×10^6^ c.f.u. ml^−1^ in fresh NB. These were then used to inoculate 96-well plates at a final density of 5×10^5^ c.f.u. ml^−1^, containing a 2-fold dilution series of CEF (1–128 µg ml^−1^) or ERT (0.05–24 µg ml^−1^). Inoculated 96-well plates were incubated at 37 °C for 24 h with no shaking, after which OD_600_ readings were recorded to assess bacterial growth. The MIC was defined as the lowest concentration of antibiotic that completely inhibits visible growth [26] which is less than 10% of the no-antibiotic control. The clinical MIC breakpoints used for interpretation were 4 mg l^−1^ for CEF [24] and 0.5 mg l^−1^ for ERT as per EUCAST guidelines [27].

### Whole-genome sequencing

Whole-genome sequencing was employed to identify mutations in each of the 24 transconjugant replicate strains (MG1655 pLL35, MG1655 pOXA-48 and MG1655 pLL35+pOXA-48) relative to the parental MG1655 strains. DNA extraction and libraries were prepared for short-read sequencing by MicrobesNG (http://www.microbesng.com), and sequencing was conducted using an Illumina NovaSeq 6000, generating 2×250 bp paired-end reads. Variants were identified with Breseq [28] using the *E. coli* MG1655 genome (GenBank accession U00096.3), pOXA-48 (GenBank accession number MT441554) and pLL35 [24] sequences as a reference. Variants identified within the parental plasmid-free *E. coli* MG1655 strain, which were also present within the transconjugants, were removed from the analysis to ensure that only newly acquired mutations were annotated.

### Functional analysis using gene knockouts (Keio collection)

Gene deletion strains from the Keio collection [29] were used to investigate the impact of loss-of-function mutations observed within genes identified to have gained mutations during acquisition of either plasmid. Parallel mutations within *ompF*, *ompR* and *envZ* were observed upon acquisition of pOXA-48; thus, pOXA-48 was conjugated into *E. coli* BW25113 *ΔompF*, *E. coli* BW25113 *ΔompR* and *E. coli* BW25113 *ΔenvZ* together with the parental *E. coli* BW25113 strain. Growth curves of all plasmid-free and pOXA-48-bearing deletion strains and the parental strain were conducted as described above in NB over 24 h at 37 °C. The resistance profiles of the Keio transconjugants to ERT were also obtained using the MIC protocols described above.

## Results

### Effects of MDR plasmids on resistance and growth

**Fig. 1.**
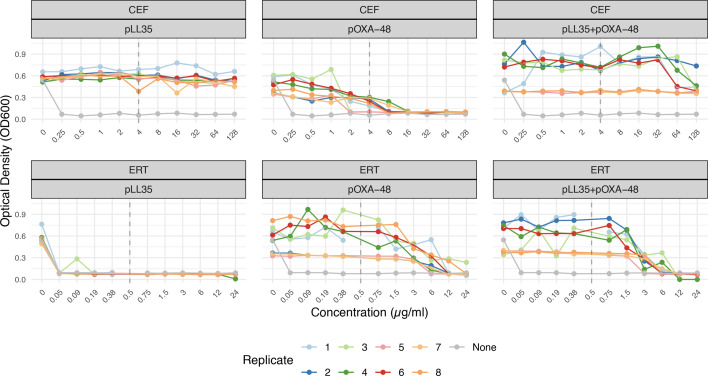
Resistance profiles of *E. coli* MG1655 isolates harbouring pLL35, pOXA-48 or both. MIC curves show OD (OD_600_) measurements for pLL35, pOXA-48 and pLL35+pOXA-48 transconjugants in the presence of increasing concentrations of CEF (top panels) and ERT (bottom panels). Each point represents the mean of 3 technical replicates for each of the 8 biological replicates (coloured lines 1–8); plasmid-free ancestral *E. coli* MG1655 control is shown in grey. Dashed vertical lines in the CEF and ERT panels indicate the concentrations of CEF (4 µg ml^−1^) and ERT (0.5 µg ml^−1^) used for transconjugant selection.

We first tested how the MDR plasmids pLL35 and pOXA-48 affected susceptibility to antibiotics, a single 24 h growth cycle following their acquisition. To do this, we determined MIC curves across concentration gradients of CEF and ERT in the newly constructed transconjugants harbouring pLL35 or pOXA-48 or both plasmids, alongside plasmid-free controls (Fig. 1). Each plasmid provided a distinct resistance profile, combining to provide high-level resistance to both antibiotics in strains carrying both plasmids (two-way ANOVA interaction F3,46=218.1, *P*<0.01). Acquiring pLL35 provided high-level resistance to CEF (MIC=>128 µg ml^−1^) without cross-resistance to ERT (MIC=0.05 µg ml^−1^). In contrast, acquiring pOXA-48 provided variable resistance to ERT (MIC range: 6–24 µg ml^−1^) and moderate cross-resistance to CEF (MIC range: 8–64 µg ml^−1^). However, there was no correlation observed between levels of resistance to ERT and CEF among the pOXA-48 transconjugants, indicating that the level of resistance to ERT does not predict the degree of cross-resistance to CEF (Spearman’s rank correlation, ρ=0, *P*-value=1). Acquiring both pLL35 and pOXA-48 provided resistance to both ERT and CEF (ERT MIC range: 3–12 µg ml^−1^; CEF MIC=>128 µg ml^−1^).

**Fig. 2.**
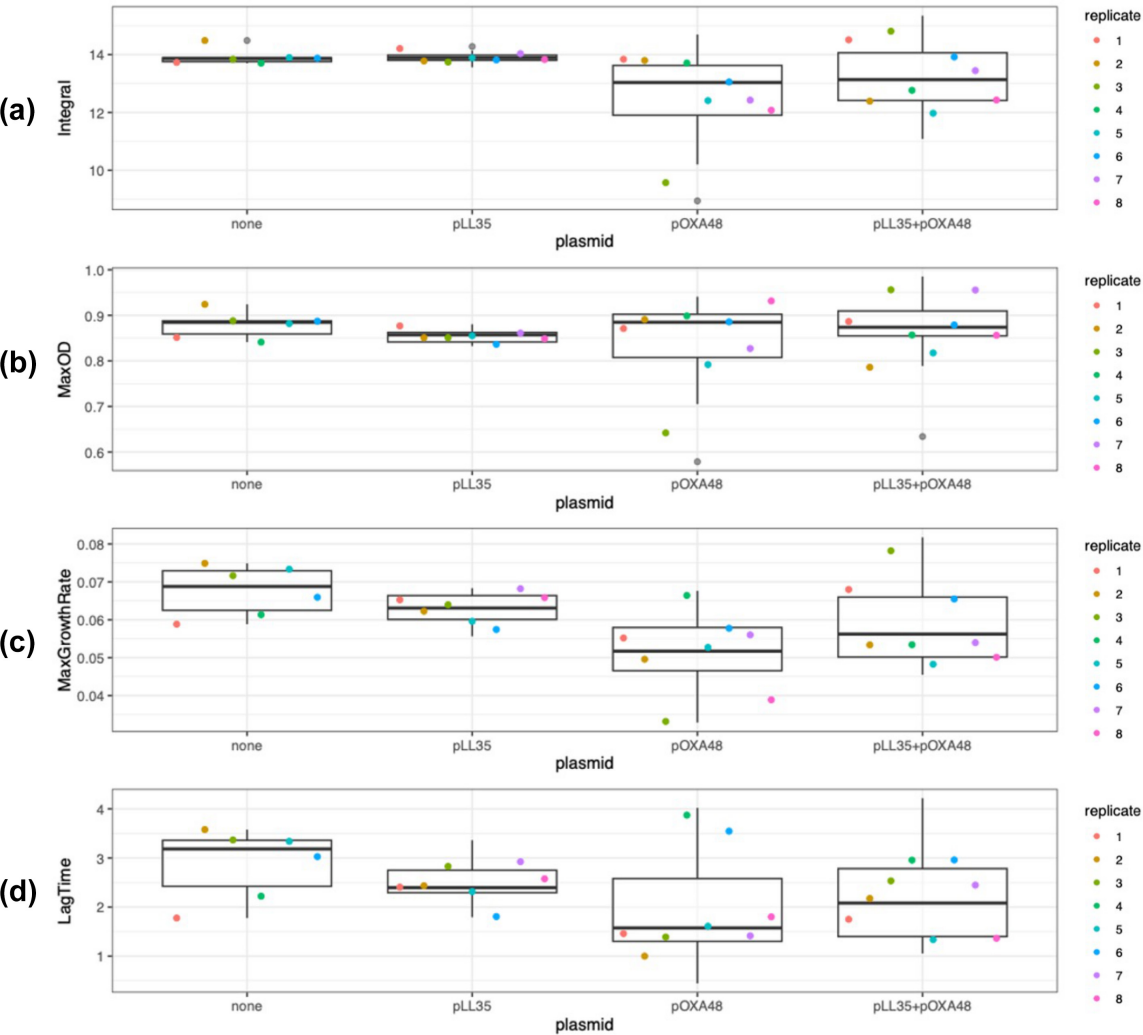
The effect of the acquisition of pOXA-48, pLL35 or both plasmids on the growth kinetics of *E. coli* transconjugants. Each coloured point represents the mean of 3 technical replicates for each biological replicate (*n*=8). Horizontal lines within the box plots represent median values, the upper and lower hinges are the 25th and 75th percentiles and the whiskers extend to observations within 1.5× the interquartile range. Panels represent the effect of plasmid(s) acquisition on (a) integral (area under the growth curve), (b) max OD, (c) max growth rate and (d) lag time for transconjugants with different plasmids, in addition to the plasmid-free control (labelled none).

We observed high variability in OD within MICs among transconjugants with pOXA-48 or both plasmids, but not when harbouring pLL35 alone. This suggested that the acquisition of pOXA-48 differentially impacted the growth of each transconjugant. To investigate these growth effects further, we obtained 24 h growth curves for all strains (Fig. S1). Transconjugants carrying pOXA-48 or both pOXA-48 plus pLL35 showed greater variance in multiple growth kinetic parameters compared to those carrying only pLL35 (Fig. 2) [MaxGrowthRate: pLL35 vs pOXA_48: F(13,15)=0.133, *P*=0.00075; pLL35 vs pLL35+pOXA_48: F(13,23)=0.143, *P*=0.00075; Integral: pLL35 vs pOXA_48: F(13,15)=0.016, *P*<0.00001; pLL35 vs pLL35+pOXA_48: F(13,23)=0.029, *P*<0.00001; LagTime: pLL35 vs pOXA_48: F(13,15)=0.195, *P*=0.00523; pLL35 vs pLL35+pOXA_48: F(13,23)=0.314, *P*=0.034]. Moreover, pOXA-48 and pLL35 differed in their impact upon the growth kinetics of *E. coli* (ANOVA: F3,56=6.647, *P*=0.000641), which was primarily driven by pOXA-48 reducing maximum growth rate (diff=−0.01644, *P*=0.0096 vs. none), maximum growth (max OD: diff=−0.03668, *P*=0.6794 vs. none) and the area under the growth curve (Integral: diff=−1.31127, *P*=0.0708 vs. none). Over a 10-day passage experiment, loss of the non-costly pLL35 was not observed in any of the transconjugants (Fig. S3), but loss of pOXA-48 was observed in 5 out of 16 of the pOXA-48 transconjugants (Fig. S3). Together, these data suggest that pOXA-48 is costlier than pLL35, but that the effects of pOXA-48 on bacterial growth vary markedly between individual transconjugants.

### Mutations obtained by transconjugants during plasmid acquisition

**Fig. 3.**
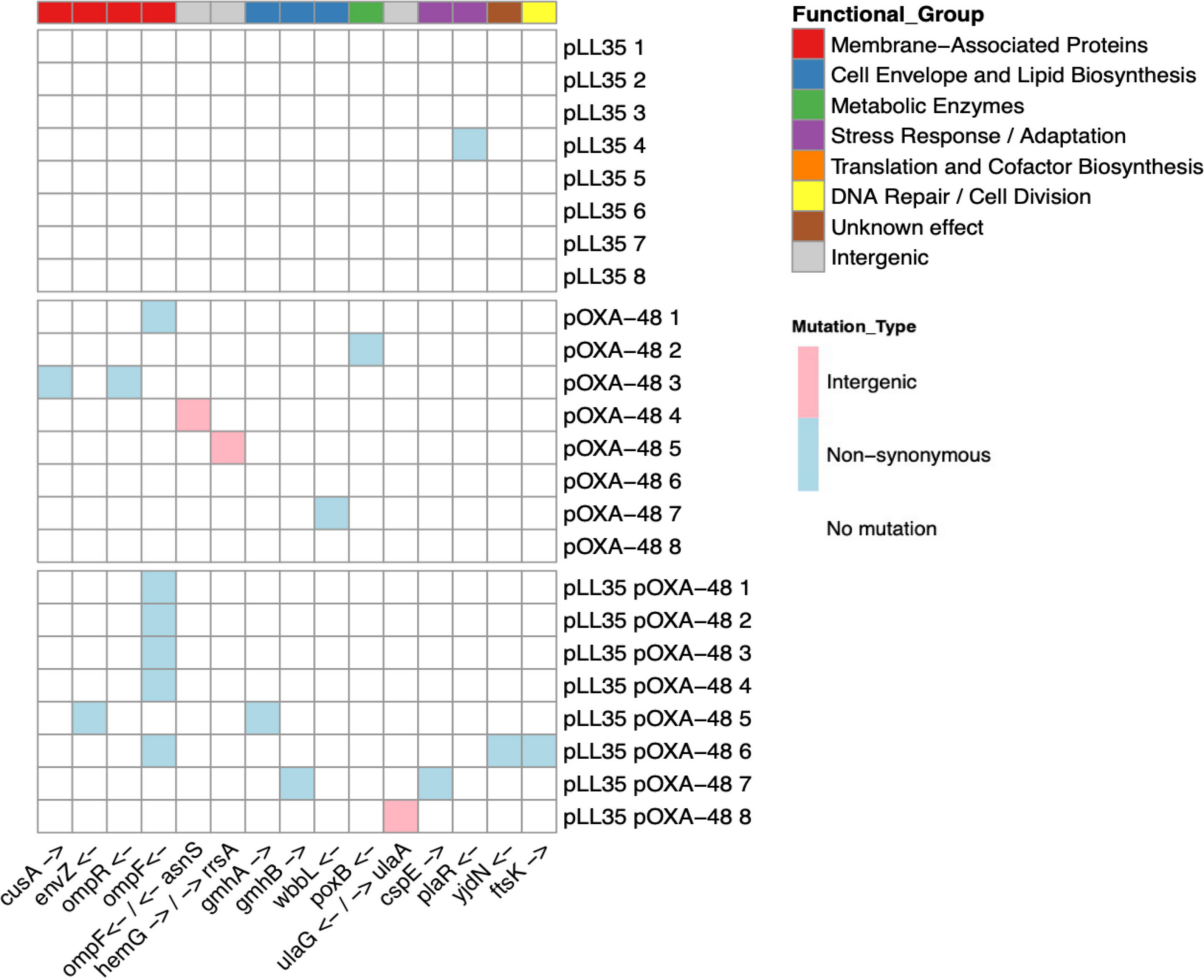
Heatmap of the distribution of chromosomal mutations across replicate *E. coli* transconjugants harbouring either pLL35, pOXA-48 or both plasmids. Each row corresponds to an independent transconjugant, and each column corresponds to a mutation locus. Pink indicates intergenic mutations, blue represents non-synonymous mutations and white denotes no variants observed. Loci are clustered into functional groups denoted by the colours at the top of the plot with grey boxes representing intergenic mutations. The arrows next to gene names represent the relative orientation of the genes (-> forward strand, <- reverse strand).

We hypothesized that the variability in growth kinetics caused by pOXA-48 could be due to differential mutations gained within each transconjugant during plasmid acquisition. To test this, we sequenced the genomes of each of the 24 newly constructed independent transconjugants. Ancestral recipients were also sequenced, and all annotated variants present in those strains were removed from the transconjugant datasets, leaving only mutations that arose post-plasmid acquisition. Fifteen of twenty-four transconjugants had at least one additional chromosomal mutation, whilst none had mutations in either plasmid (Fig. 3). Whereas eight out of eight transconjugants with both plasmids and six out of eight transconjugants with pOXA-48 alone had gained additional mutation(s), only one out of eight transconjugants with pLL35 did (Table S3). By far the most commonly mutated gene was *ompF*, encoding an outer membrane pore-forming protein associated with antibiotic permeability [22]. Mutations were detected in five transconjugants with both plasmids – four resulting in frameshifts and one in a premature stop codon – and in one transconjugant carrying only pOXA-48, resulting in a frameshift. Notably, three additional mutations were observed in other transconjugants carrying either pOXA-48 or both plasmids that are likely to affect the expression of *ompF*. These included both genes of the *envZ-ompR* two-component system regulating *ompF* having missense mutations [30], and an intergenic mutation located 50 bp from the ompF transcription start site. In total, 9 out of 16 transconjugants containing pOXA-48 had gained additional mutations affecting *ompF* or its regulation. No correlation was observed between pOXA-48 copy number and MIC for ERT for transconjugants with pOXA-48 alone or with pLL35 (Spearman’s pOXA-48 ρ=0.18, *P*=0.50, pOXA-48+pLL35 ρ=0.25, *P*=0.55, Fig. S4 and Table S5).

**Fig. 4.**
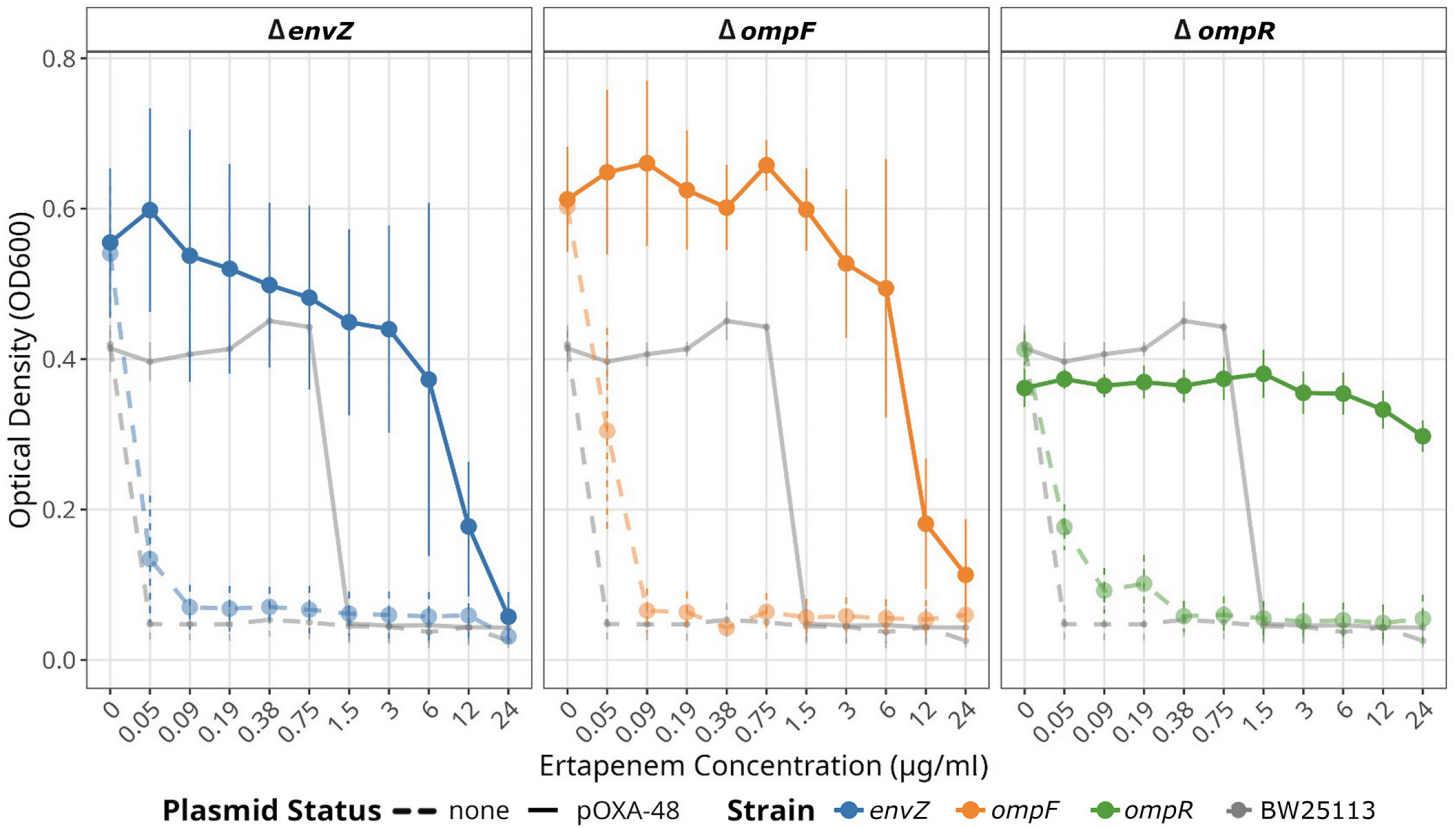
ERT resistance profiles of *E. coli* BW25113 and knockout mutants with and without the pOXA-48 plasmid. Each point represents the mean growth (OD_600_) of three replicates per strain at each drug concentration, in the presence of increasing ERT concentration. The plot is faceted by gene knockout; dashed lines show growth with no plasmid, and solid lines show growth harbouring pOXA-48. Grey lines in each panel show the growth of WT *E. coli* BW25113 for reference.

Given the well-established role of OmpF in cellular antibiotic permeability [22,30, 31], we next tested how loss of *ompF*, *ompR* or *envZ* altered ERT resistance with or without pOXA-48 using single-gene knockout strains for each gene from the Keio library (Fig. 4). Whereas none of these mutations increased ERT resistance without the plasmid (effect of chromosomal mutation F(3,8)=69.06, *P*=0.42), each was strongly synergistic with pOXA-48, amplifying the plasmid-mediated ERT resistance (interaction between chromosomal mutation and pOXA-48 F(3,8)=69.06, *P*<0.001), with Δ*ompR* having the largest synergistic effect (interaction effect size, Δ*envZ=*3.8, Δ*ompF*=5.1 and Δ*ompR*=6.1 AUC units; Fig. S5 and Table S4).

**Fig. 5.**
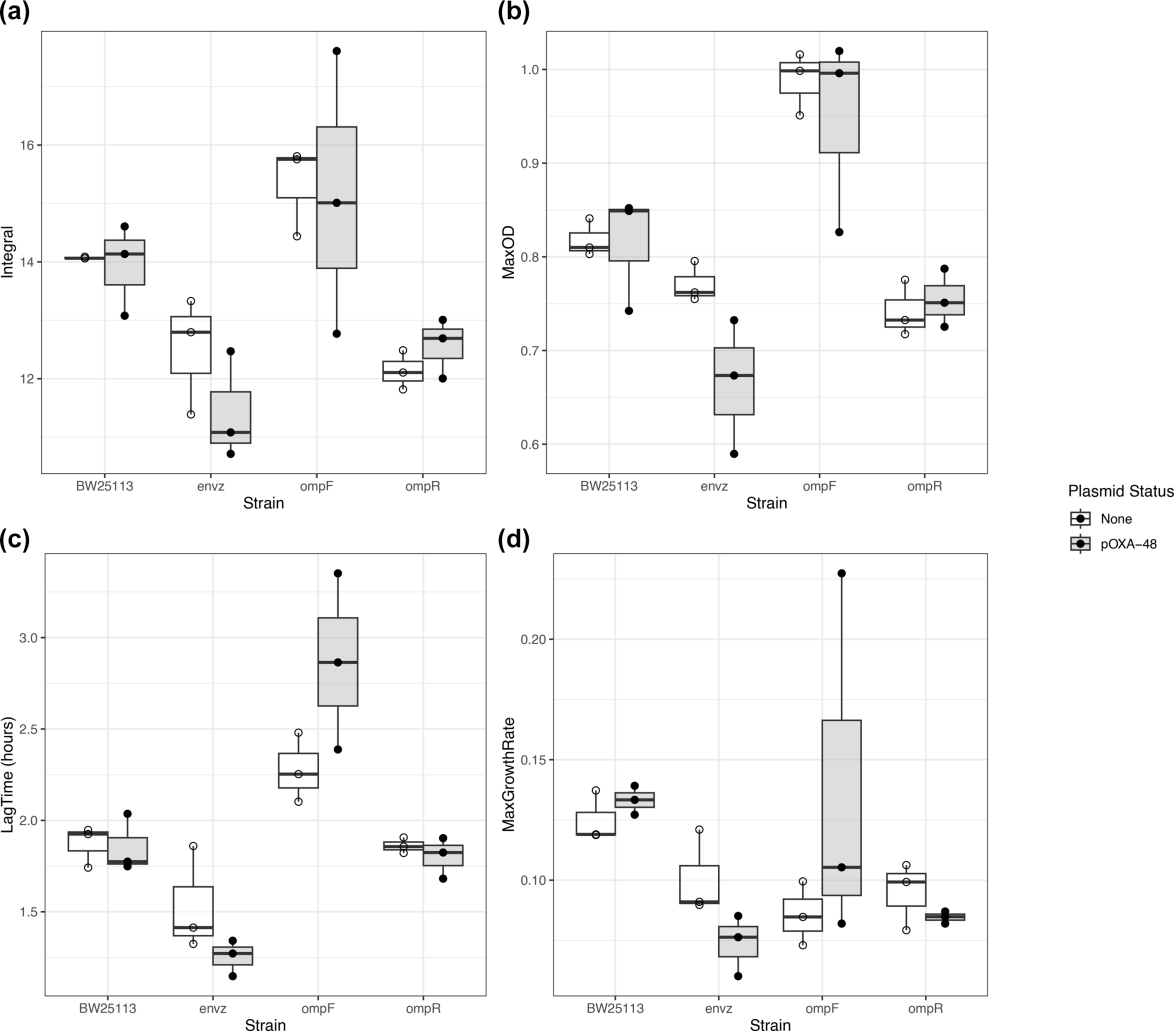
The effect of the acquisition of pOXA-48 on the growth kinetics of the knockout strains (Δ*envZ*, Δ*ompF* and Δ*ompR*) and WT *E. coli* BW25113. Grey-filled boxplots represent plasmid-bearing strains, and white (unfilled) boxplots represent their plasmid-free counterparts. Each point represents the mean of three technical replicates for each of three biological replicates. Horizontal lines within the box plots indicate median values; the upper and lower hinges are the 25th and 75th percentiles, and the whiskers extend to observations within 1.5× the interquartile range. Panels (a–d) show the effect of pOXA-48 acquisition on growth parameters by comparing plasmid-free and plasmid-bearing versions of knockout strains: (a) integral (AUC), (b) maximum OD₆₀₀, (c) lag time and (d) maximum growth rate.

Next, using 24 h growth curves (Fig. S2), we tested the effect of these chromosomal mutations on pOXA-48 fitness costs (Fig. 5). We observed no compensatory effect of losing any of these genes on the growth of plasmid carriers (ANOVA max growth rate, F1,52=0.511, *P*=0.47805); indeed, loss of *envZ* strongly impaired growth both with and without pOXA-48 (post-hoc Tukey tests, envZ:BW25113, *P*=0.0013; envZ pOXA-48:BW25113 pOXA-48, *P*=0.0046), whereas loss of *ompF* increased growth regardless of plasmid carriage (post-hoc Tukey tests, ompF pOXA-48:ompF none, *P*=0.0490). These effects are consistent with the specific mutations observed in the transconjugants – the frameshift and nonsense mutations in *ompF* likely result in loss of functions similar to our knockout strains, whilst the missense mutation in *envZ* (I86S) may have more subtle effects than total deletions (Table S3). Together, these patterns suggest that mutation of *ompF* and its regulators in transconjugants was primarily favoured due to the synergistic effects on ERT resistance, rather than compensatory effects on fitness.

## Discussion

We show that additional chromosomal resistance mutations occurring during the formation (or outgrowth) of transconjugant colonies facilitated the acquisition of pOXA-48 but not during the acquisition of pLL35. These mutations affected the outer membrane protein OmpF, or its regulators *ompR* or *envZ*. OmpF is known to control antibiotic permeability, including of carbapenems [30,32]. Loss of *ompF* or either regulator did not alone substantially increase ERT resistance, but instead these chromosomal mutations acted synergistically with the OXA-48 ARG encoded by pOXA-48 to greatly increase ERT resistance in plasmid carriers. None of these mutations were compensatory for the plasmid, suggesting that it was their effect on resistance level rather than any compensatory effect upon fitness that led to their enrichment in pOXA-48 transconjugants. Overall, our results highlight how chromosomal resistance and acquired ARGs can have strong epistatic effects on resistance level, here acting synergistically to provide greater than additive levels of resistance to ERT and facilitating the acquisition of MDR plasmids in antibiotic environments.

Previous studies report synergy between chromosomal and plasmid-encoded resistance. Through a synergistic pairing of reduced tetracycline influx, via the loss of function of *ompF*, and efflux via a plasmid-encoded tetracycline efflux pump, *E. coli* can modulate resistance, increasing the level of resistance whilst minimizing costs [19]. Synergy between antibiotic inactivation and influx has also been reported; the trace catalytic activity of the plasmid-encoded ESBL CTX-M-15 and reduced drug influx has previously been shown to promote the evolution of carbapenem resistance through the altered expression of porins leading to increased resistance to ERT [30]. Coupling of *β*-lactamase production and porin inactivation has also been observed within non-carbapenemase-producing CRE isolates from patients with bacteraemia, resulting in high-level carbapenem resistance [33]. Similarly, transconjugants receiving plasmids encoding New Delhi metallo-*β*-lactamase 1 (*bla*_NDM-1_) rapidly acquired mutations in *ompR* altering the expression of *ompC* and *ompF*, when selected for using the carbapenem meropenem [34]. In our case, synergy arises between a plasmid-encoded carbapenemase OXA-48, which alone provides resistance to ERT, and mutations which act to lower antibiotic permeability. Together, these are likely to reduce the rate of periplasmic accumulation of antibiotic [35], resulting in a reduction of the enzymatic activity required to overcome lethal concentrations of carbapenems [6], thus providing high-level resistance. There is mounting evidence supporting the synergistic interaction between influx and enzymatic inactivation; combining pOXA-48 with ΔOmpK35/36 in *K. pneumoniae* increases meropenem and cephalosporin MICs by up to 128-fold [36]. Likewise, carbapenem MICs increase between 8- and 32-fold in *K. pneumoniae* when CTX-M-14 or CMY-2 *β*-lactamases are combined with ΔompK35/36 [37]. Clinical Enterobacteriaceae with decreased OmpF/OmpC expression combined with pOXA-48 carriage have >100-fold MIC increases relative to either mechanism alone [38].

Rapid compensatory evolution during transconjugant formation can stabilize plasmids [21]. Here, we demonstrate another way in which rapid evolutionary dynamics can facilitate plasmid-mediated HGT, specifically by chromosomal resistance mutations enhancing the fitness benefits of the plasmid-encoded ARGs under antibiotic selection. Such rapid evolutionary processes may be important for understanding plasmid dynamics in environments with strong antibiotic selection for plasmid-encoded ARGs, and especially where these ARGs provide only marginal resistance, as observed here with ERT and pOXA-48. In contrast, when plasmid-encoded resistance mechanisms provide very high-level resistance, such as CTX-M-15 with CEF, no additional resistance mechanisms were required for the stable plasmid transfer during CEF exposure. Additional chromosomal mutations were also required during the acquisition of both pLL35 and pOXA-48 under multidrug treatment, indicating that there was no synergy between the two plasmids in resistance to ERT.

Our findings are consistent with clinical observations, where pOXA-48–producing Enterobacterales frequently exhibit mutations in genes encoding outer membrane protein or their regulators, such as *ompR* and *ompF*, that reduce outer membrane permeability [30,31]. For instance, clinical isolates of *K. pneumoniae* with alterations in *ompC* and *ompF* demonstrate significantly reduced susceptibility to carbapenems, including ERT and meropenem [22]. These chromosomal resistance mutations, together with plasmid-borne beta-lactamases, create highly resistant clinical strains, reducing treatment options. Moreover, synergistic epistasis between chromosomal and plasmid-encoded resistance determinants is likely to complicate the prediction of antibiotic resistance level from genomic data.

## Supplementary material

10.1099/mic.0.001599 Uncited Supplementary Material 1.

10.1099/mic.0.001599 Uncited Supplementary Material 2.

## Abbreviations

ARGs: antimicrobial resistance genes
AUC: area under the curve
CEF: cefotaxime
CRE: carbapenem-resistant Enterobacteriaceae
ERT: ertapenem
ESBL: extended-spectrum *β*-lactamase
HGT: horizontal gene transfer
MDR: multidrug-resistant
MGEs: mobile genetic elements
NB: Nutrient Broth
