# Supplementary material for: Chromosomal resistance mutations facilitate acquisition of multidrug-resistant plasmids in Escherichia coli
Source: Microbiology (Reading). 2025 Sep 25;171(9):001599. doi: 10.1099/mic.0.001599 (PMC13293288; doi:10.1099/mic.0.001599)
Supplement: Uncited Supplementary Material 1. [file mic-171-01599-s001.pdf]

SUPPLEMENTARY MATERIAL_FIGURES

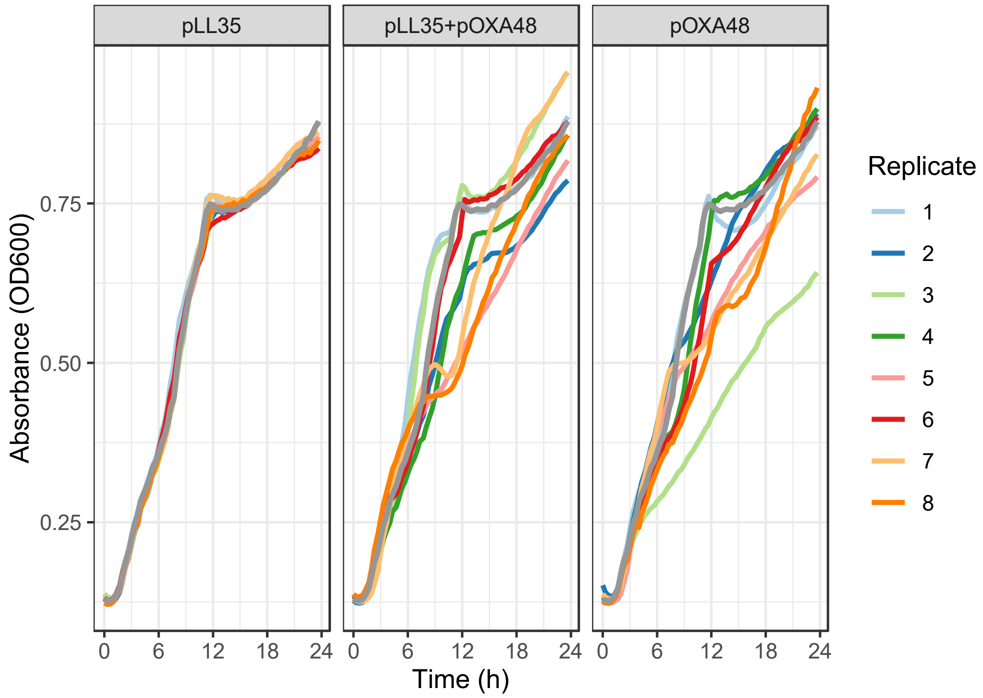

Figure S1: Growth curve of 8 transconjugants per plasmid type with OD600 plotted against time in hours. The grey line overlayed is the reference plasmid-free ancestral plasmid. Each of the coloured lines is the average of 3 replicates of each individual replicate in the three plasmid groups.

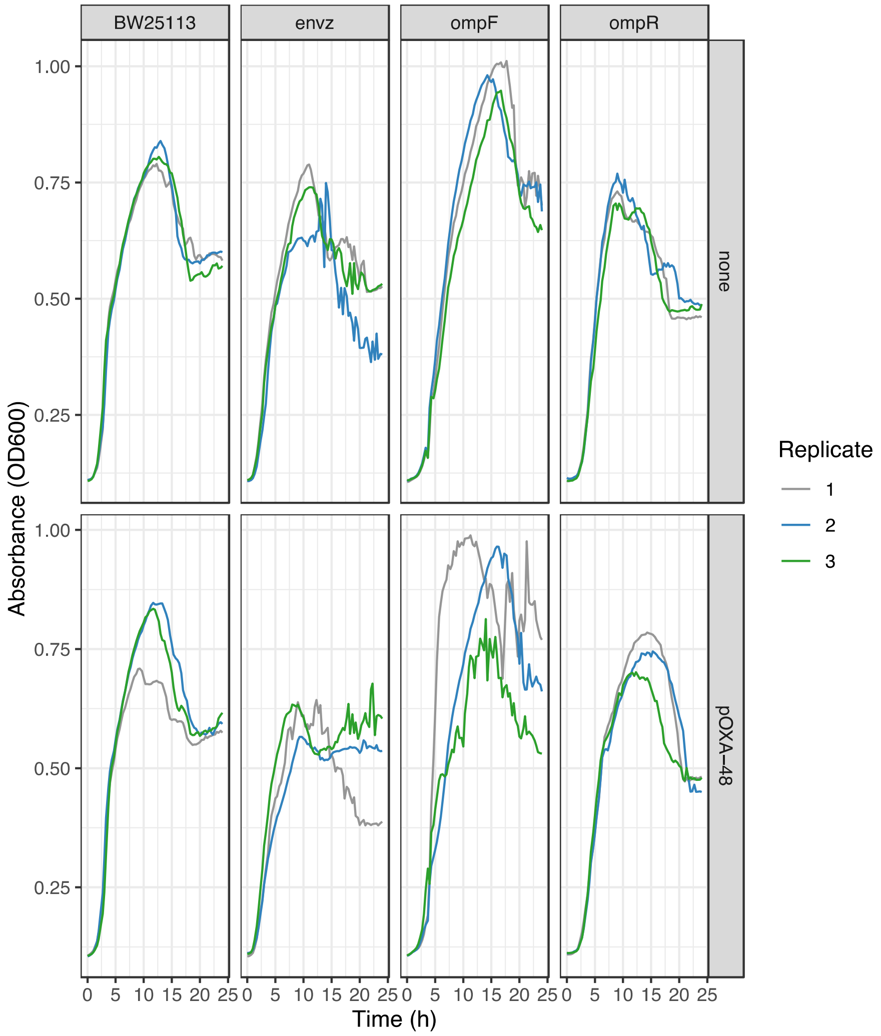

Figure S2: Growth Curves of Keio Knockouts and BW25113 Strains with and without pOXA-48 Plasmid. Figure shows the 24-hour growth curves (OD600 absorbance) of E. coli BW25113 and Keio knockout strains (*envZ*, *ompF*, *ompR*) with and without the pOXA-48 plasmid. The top panel presents growth curves for plasmid-free strains, and the bottom panel shows strains carrying the pOXA-48 plasmid. Each plot represents a specific strain, with each line being the mean of three 3 technical replicates. The Y-axis represents the optical density at 600 nm (OD600), which serves as a measure of cell growth, and the X-axis represents the growth time in hours.

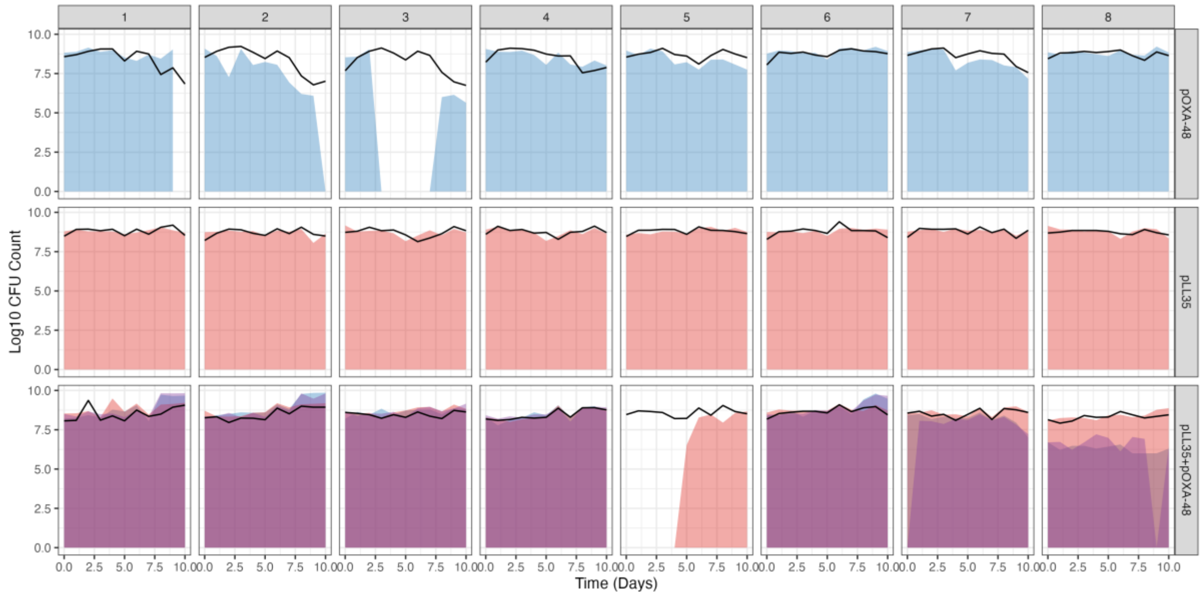

Figure S3: Time-course of plasmid stability in *Escherichia coli* MG1655 over ten days. Faceted ribbon-and-line plots display Log_{10} CFU·mL^{-1} counts for eight independent biological replicates (columns 1–8) under three plasmid backgrounds (rows A–C). The top row shows pOXA-48 transconjugants, middle row pLL35 transconjugants, and bottom row dual pLL35+pOXA-48 transconjugants. Colored ribbons represent the absolute count of plasmid-bearing subpopulations on selective agar: blue for ertapenem selection, red for cefotaxime selection, and purple for combined ertapenem + cefotaxime selection. The solid black line traces total viable counts on non-selective nutrient agar. The x-axis denotes days post-inoculation (0–10 days) and the y-axis denotes Log_{10} CFU·mL^{-1}. Zero or counts bellow threshold for detection are plotted as $\mathrm{Log}_{10} = 0$.

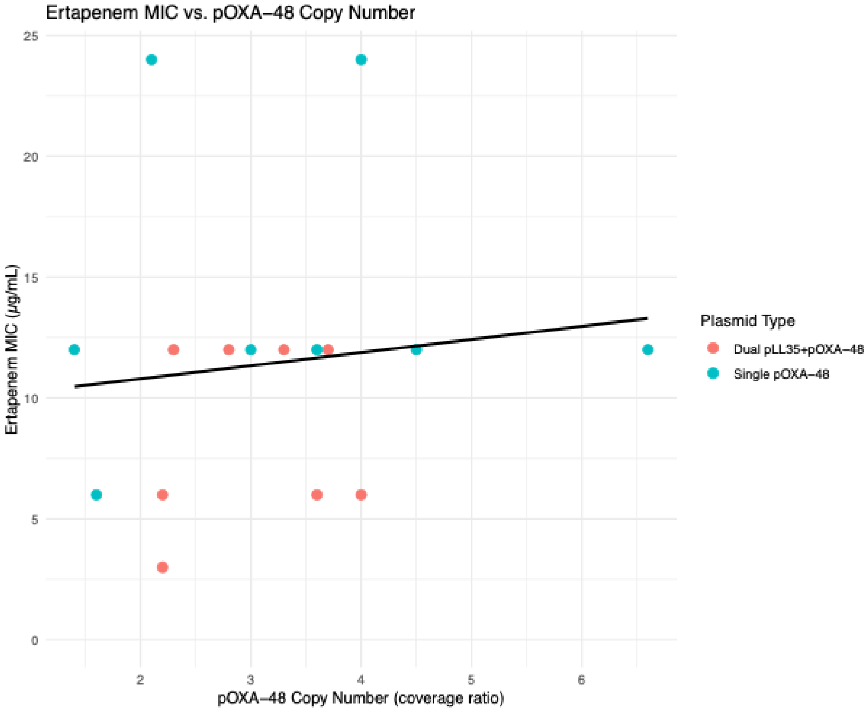

Figure S4: Scatterplot of ertapenem MIC vs pOXA-48 copy number in transconjugant strains. Single-plasmid isolates (pOXA-48 only) are shown as blue dots while dual-plasmid isolates (pLL35 + pOXA-48) are shown as red dots. The solid black line is the fitted linear regression, and its near-zero slope indicates that increases in pOXA-48 copy number does not equate to measurable, consistent increases in ertapenem MIC. This lack of a positive slope illustrates that copy number alone cannot account for the variation in resistance levels observed.

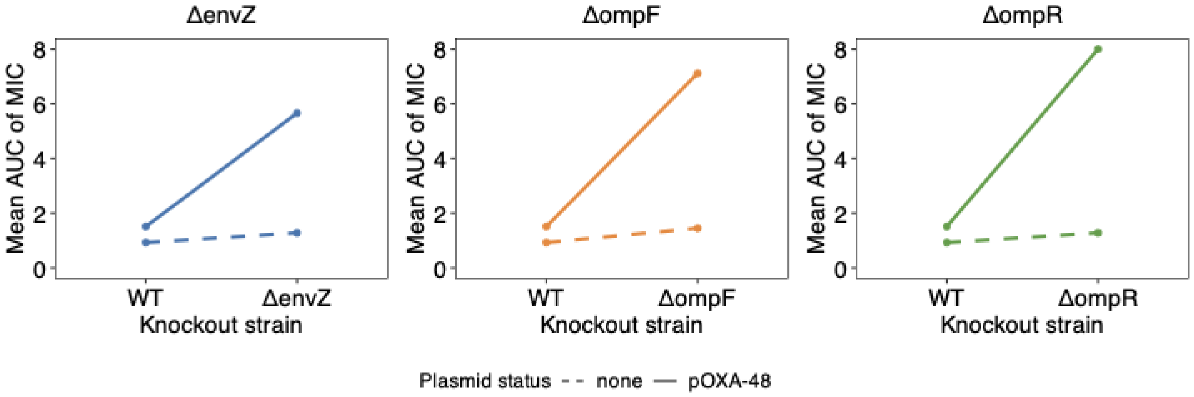

Figure S5. Interaction plot showing the strong synergistic interaction between the presence of chromosomal mutations Δ*envZ,* Δ*ompF* and Δ*ompR* and the presence of the pOXA-48 plasmid upon resistance to ERT, calculated using AUC of MIC curves (Figure 4). The dashed line shows the change in AUC between strains containing the deletions and their isogenic WT parental strains in the absence of the pOXA-48 plasmid and the solid lines show the change in presence of the pOXA-48 plasmid. There is a significant interaction between slopes of the lines (interaction between chromosomal mutation and pOXA-48 $F(3,8) = 69.06$, $p < 0.001$), with Δ*ompR* having the largest synergistic effect (interaction effect size, Δ*envZ* = 3.8, Δ*ompF* = 5.1 and Δ*ompR* = 6.1).
